# Alteration of plasma von Willebrand factor in the treatment of retinal vein occlusion with cystoid macular edema

**DOI:** 10.1371/journal.pone.0264809

**Published:** 2022-09-22

**Authors:** Hiromasa Hirai, Mariko Yamashita, Masanori Matsumoto, Takeyuki Nishiyama, Daishi Wada, Naoko Okabe, Yutaro Mizusawa, Hironobu Jimura, Tetsuo Ueda, Nahoko Ogata

**Affiliations:** 1 Department of Ophthalmology, Nara Medical University, Kashihara, Japan; 2 Department of Ophthalmology, Nara City Hospital, Nara, Japan; 3 Department of Blood Transfusion Medicine, Nara Medical University, Kashihara, Japan; Yamagata University Faculty of Medicine: Yamagata Daigaku Igakubu Daigakuin Igakukei Kenkyuka, JAPAN

## Abstract

Retinal vein occlusion (RVO) is a major retinal disease caused by venous thrombosis. Although several studies have proposed an association between venous thrombosis and von Willebrand factor (VWF), the association between RVO and VWF remains unclear. We aimed to investigate the association between RVO and VWF and the alteration of VWF levels under anti-vascular endothelial growth factor (VEGF) treatment. We enrolled 55 patients with RVO involved cystoid macular edema. They received intravitreal injection of anti-VEGF drugs, either ranibizumab or aflibercept. We examined the clinical data and measured plasma VWF antigen and a disintegrin and metalloproteinase with a thrombospondin type 1 motif, member 13 (ADAMTS13) activity to identify variabilities during treatment. At baseline, there was no significant difference between the RVO group and age-matched controls in both VWF antigen and ADAMTS13 activity levels, but ADAMTS13 activity was significantly lower in central RVO than in branch RVO (*P* = 0.015). In branch RVO, VWF antigen was negatively correlated with central choroidal thickness (r = −0.51, *P* < 0.001). In branch RVO after anti-VEGF treatment, VWF antigen levels decreased significantly from 134% at baseline to 109% at 1 day (*P* = 0.002) and 107% at 1 month (*P* = 0.030) after treatment. In contrast, ADAMTS13 activity showed no significant difference during this period. In branch RVO at 1 month after treatment, VWF antigen was negatively correlated with central choroidal thickness (r = −0.47, *P* = 0.001). Our findings suggest an association between VWF and central choroidal thickness in patients with branch RVO, thus the measurement of VWF may be useful for evaluating disease activity and prognosis.

## Introduction

Von Willebrand factor (VWF), a large glycoprotein synthesized and secreted from vascular endothelial cells, acts as a bridging molecule to adhere platelets to injured vessels and stabilize coagulation factor VIII [1]. VWF is specifically cleaved by disintegrin and metalloproteinase with a thrombospondin type 1 motif, member 13 (ADAMTS13) [2]. The balance between VWF and ADAMTS13 maintains the hemostatic coagulation function of the whole body. The release of VWF increases with endothelial cell damage, and elevated plasma VWF levels are usually observed in patients with hypertension and atherosclerosis [3–5]. Karaca et al. reported an association between plasma VWF and the severity of hypertensive retinopathy [6]. In other ocular diseases, elevated plasma VWF antigen levels have been reported in age-related macular degeneration, and multimeric VWF has been found in pachychoroid neovasculopathy [7–9].

Several studies have also reported that increased plasma VWF levels are associated with thrombotic complications [10–12]. This association includes not only atherothrombotic diseases (myocardial infarction or ischemic stroke) but also venous thrombosis. Edvardsen et al. recently suggested an association between VWF and venous thrombosis, including deep vein thrombosis and pulmonary embolism [12].

Retinal vein occlusion (RVO) is a major retinal disease that causes retinal hemorrhage and severe vision loss [13,14]. Vision loss in RVO is mainly due to cystoid macular edema. RVO is a multifactorial disease with several risk factors, including age, hypertension, arteriosclerosis, diabetes mellitus, dyslipidemia, high blood viscosity, and thrombosis [14,15]. RVO can be classified into two types: branch RVO and central RVO. In branch RVO, although partial retinal ischemia may occur, blood perfusion throughout the retina is generally maintained [16]. Alternatively, the entire retina can become severely ischemic in central RVO, leading to severe vision loss and neovascular glaucoma [17]. Central RVO usually has a worse prognosis than branch RVO. In patients with RVO, understanding the retinal circulatory status is important for controlling the disease.

Intravitreal injection of anti-vascular endothelial growth factor (VEGF) drugs has been widely used for the treatment of RVO with cystoid macular edema. However, cystoid macular edema often recurs and requires additional injections. The systemic side effects of anti-VEGF vitreous injection remain controversial. Several studies have reported thromboembolic events, including myocardial infarction and ischemic stroke [18,19]. In addition, anti-VEGF antibodies enter the systemic circulation, decrease VEGF levels, and affect other cytokines in the plasma [20]. Previously, we demonstrated that VWF antigen levels decreased after intravitreal aflibercept injection in patients with age-related macular degeneration [8].

Although RVO is caused by venous thrombosis, few clinical studies have examined the association between VWF and RVO; and these studies have reported different results. Thus, the levels of VWF in RVO remain controversial [21–23]. Furthermore, no study has investigated whether plasma VWF is influenced by anti-VEGF vitreous injection in patients with RVO. In this study, we aimed to investigate the involvement of VWF in the pathogenesis of RVO and its variability after anti-VEGF injection.

## Methods

This prospective study was conducted at Nara Medical University Hospital, Kashihara City, Nara Prefecture, Japan from June 2014 to March 2020. The study protocol was approved by the Medical Research Ethics Committee of Nara Medical University and followed the Declaration of Helsinki. We confirmed that all methods were performed in accordance with the relevant guidelines and regulations. All patients provided written informed consent for participation in the study. We included consecutive patients who provided consent within the research period approved by the Ethics Committee. Finally, we enrolled 55 patients diagnosed with RVO with cystoid macular edema between the ages of 51 and 91 years and 96 age-matched controls (patients before cataract surgery). Patients who had severe systemic complications or who did not receive anti-VEGF drugs, i.e., selected other treatments, were excluded.

RVO was diagnosed based on ophthalmological findings, slit lamp examination, fundus examination, optical coherence tomography, wide-angle fundus photography, and fluorescein angiography. We also examined best-corrected visual acuity (BCVA, LogMAR unit), central retinal thickness (CRT), and central choroidal thickness (CCT). The pathological classification (branch RVO or central RVO) was judged independently by two researchers based on the examinations.

All patients received intravitreal injections of anti-VEGF drugs, either ranibizumab (Lucentis; Novartis International AG, Basel, Switzerland) or aflibercept (Eylea; Bayer HealthCare Pharmaceuticals, Berlin, Germany) at a dose of 2.0 mg/0.05 mL. The drugs were selected by the attending physicians. The intravitreal injection was administered 3.5–4.0 mm posterior to the corneal limbus.

Whole blood was collected by venipuncture of the anterior arm into a tube containing 3.8% trisodium citrate (1:9). Blood samples were collected four times: before treatment (first visit), 1 day after treatment, 1 week after treatment, and 1 month after treatment. All plasma samples were stored at −80°C and thawed at 37°C prior to examination.

Plasma VWF antigen levels were measured by sandwich enzyme-linked immunosorbent assay (ELISA) using a rabbit anti-human VWF polyclonal antiserum (DAKO, Glostrup, Denmark) [24]; plasma ADAMTS13 activity, by chromogenic ADAMTS13 activity ELISA (Kainos, Tokyo, Japan) [25]. We defined the 100% reference value as the amount of VWF antigen and ADAMTS13 activity in pooled normal plasma from 20 volunteers (10 men and 10 women) aged between 20 and 40 years.

All statistical analyses were performed using EZR (Saitama Medical Center, Jichi Medical University, Saitama, Japan), a graphical user interface for R (The R Foundation for Statistical Computing, Vienna, Austria) [26]. We used T-tests to compare two continuous variables (such as age) and Fisher’s exact tests to compare the proportions of categorical variables (such as sex) between the groups. We also used the Friedman and Wilcoxon signed-rank tests to compare the four continuous variables (VWF antigen). Correlations were evaluated using Pearson’s correlation coefficient. The threshold for significance was set at *P* < 0.05.

## Results

The characteristics of the patients with RVO and controls are summarized in Table 1. In patients with RVO, the median age was 73.0 years, and 33 (60%) were male. Twenty-seven patients (49%) had hypertension and twenty-one (38%) had smoking experience. There was no significant difference in VWF antigen and ADAMTS13 activity between the RVO group and the controls (*P* = 0.70 and *P* = 0.22, respectively). We also examined the baseline characteristics of patients with RVO classified according to RVO type (branch RVO and central RVO). Both CRT and CCT were significantly thicker in central RVO than in branch RVO (*P* = 0.013 and *P* = 0.045, respectively). The number of dyslipidemia cases was also significantly higher in central RVO than in branch RVO (*P* = 0.004). Although VWF antigen was not significant between the two groups (*P* = 0.18), ADAMTS13 activity was significantly lower in central RVO than in branch RVO (*P* = 0.015). In other categories, there were no significant differences between the two groups.

**Table 1:** Basic characteristics of RVO patients and controls.

	Total RVO (n = 55)	Control (n = 96)	*P* value	Branch RVO (n = 43)	Central RVO (n = 12)	*P* value
Age, median (IQR)	73.0 (65.0–79.0)	74.0 (69.0–80.0)	0.18^†^	73.0 (66.0–79.5)	72.0 (64.0–74.3)	0.33^†^
Sex (male), n (%)	33 (60)	52 (54)	0.50^‡^	23 (53)	10 (83)	0.10^‡^
BCVA, median (IQR),	0.40 (0.15–0.52)	―		0.30 (0.15–0.52)	0.40 (0.28–0.57)	0.19^†^
CRT (μm), median (IQR)	477 (357–614)	―		440 (343–586)	574 (491–747)	0.013^†^
CCT (μm), median (IQR)	238 (197–264)	―		235 (180–259)	257 (214–298)	0.045^†^
Hypertension, n (%)	27 (49)	―		20 (47)	7 (58)	0.53^‡^
Dyslipidemia, n (%)	13 (24)	―		6 (14)	7 (58)	0.004^‡^
Diabetes, n (%)	6 (11)	―		4 (9)	2 (17)	0.60^‡^
Cardiovascular disease, n (%)	8 (15)	―		6 (14)	2 (17)	1^‡^
Smoking status, n (%)						
Past or Current	21 (38)	29 (30)	0.37^‡^	17 (40)	4 (33)	0.75^‡^
Never	22 (40)	67 (70)	-	17 (40)	5 (42)	-
Unknown	12 (22)	0 (0)	-	9 (21)	3 (25)	-
VWF (%), median (IQR)	134 (92–173)	130 (109–167)	0.70^†^	139 (107–175)	92 (60–122)	0.18^†^
ADAMTS13 (%), median (IQR)	45 (33–61)	54 (45–63)	0.22^†^	51 (38–69)	34 (29–39)	0.015^†^
Blood Type, n (%)						
Type O	18 (33)	32 (33)	1^‡^	12 (28)	6 (50)	0.18^‡^
Type A	23 (42)	34 (35)	0.49^‡^	19 (44)	4 (33)	0.74^‡^
Type B	9 (16)	16 (17)	1^‡^	9 (21)	0 (0)	0.18^‡^
Type AB	5 (9)	14 (15)	0.45^‡^	3 (7)	2 (17)	0.30^‡^

Abbreviations: RVO, retinal vein occlusion; IQR, interquartile range; BCVA, best-corrected visual acuity (LogMAR unit); CRT, central retinal thickness; CCT, central choroidal thickness; VWF, von Willebrand factor.

^†^T-test.

^‡^Fisher’s exact test.

We also assessed the blood type because blood type O has the lowest plasma VWF antigen levels [27]. However, there was no difference in blood type between the two groups.

**Fig 1:**
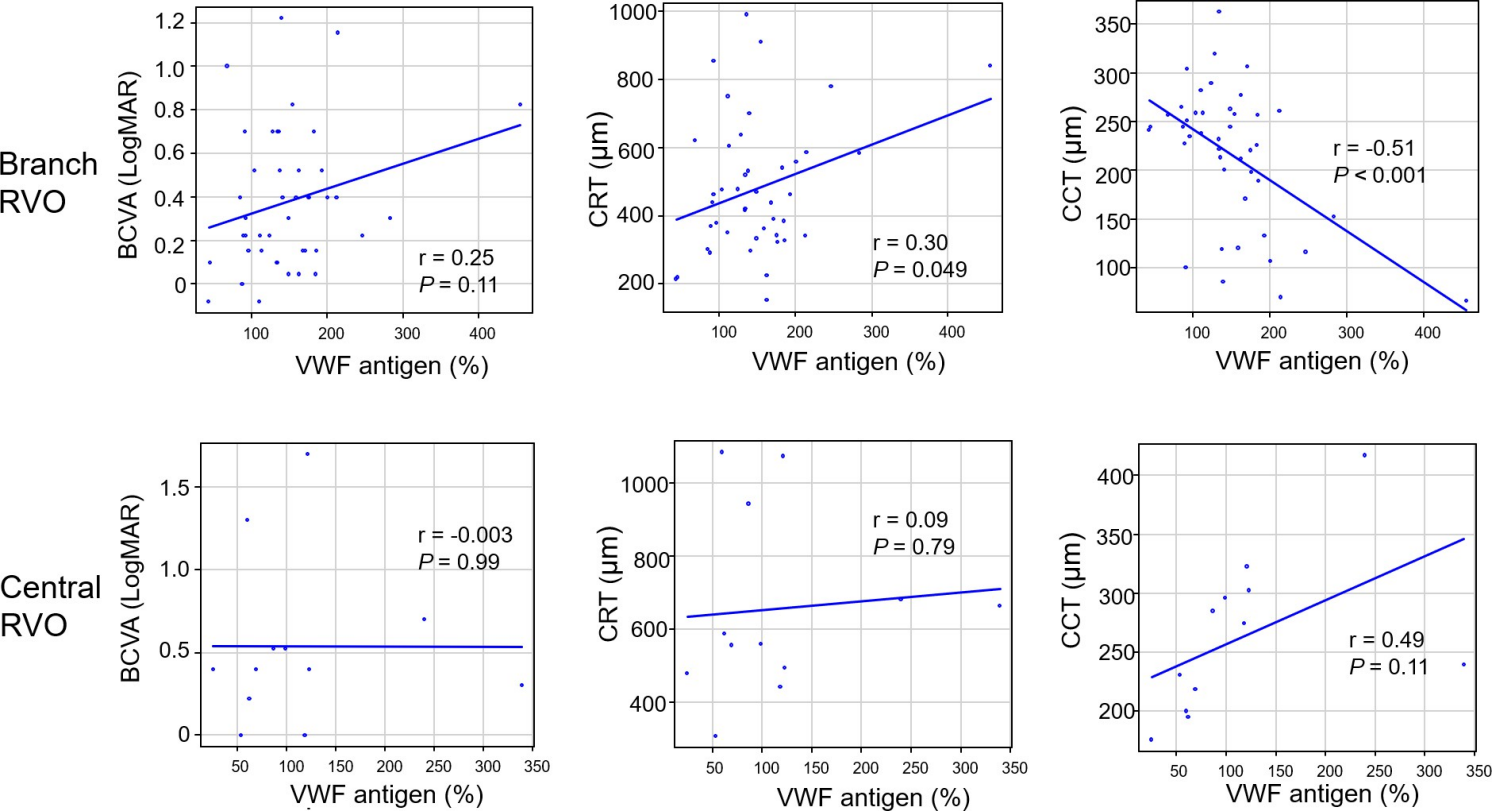
The correlation between VWF antigen and parameters (BCVA, CRT, and CCT) at baseline. In each graph, the horizontal axis presents the VWF antigen (%). BCVA, best-corrected visual acuity (LogMAR unit); CRT, central retinal thickness; CCT, central choroidal thickness; RVO, retinal vein occlusion.

Fig 1 shows the correlation between VWF antigen and each parameter at baseline. In branch RVO, VWF antigen was positively correlated with CRT (r = 0.30, *P* = 0.049) but negatively correlated with CCT (r = −0.51, *P* < 0.001). In central RVO, no correlations were found in any of the categories.

Table 2 shows the basic characteristics of patients with RVO classified according to the selected anti-VEGF drugs. There were no significant differences between the two groups.

**Table 2:** Basic characteristics of patients with RVO classified according to anti-VEGF drugs.

	Aflibercept (n = 28)	Ranibizumab (n = 27)	*P* value
Age, median (IQR)	74.0 (66.0–79.0)	72.0 (63.5–78.0)	0.78^†^
Sex (male), n (%)	15 (54)	18 (67)	0.41^‡^
RVO type (Branch RVO / Central RVO)	21 / 7	22 / 5	0.75^‡^
BCVA, median (IQR)	0.22 (0.14–0.52)	0.39 (0.26–0.61)	0.17^†^
CRT (μm), (IQR)	456 (358–626)	481 (371–586)	1^†^
CCT (μm), (IQR)	233 (199–257)	251 (187–278)	0.58^†^
VWF (%), median (IQR)	118 (82–164)	139 (111–183)	0.54^†^
ADAMTS13 (%), median (IQR)	40 (31–53)	51 (34–69)	0.67^†^

Abbreviations: IQR, interquartile range; BCVA, best-corrected visual acuity (LogMAR unit); CRT, central retinal thickness; CCT, central choroidal thickness; VWF, von Willebrand factor; RVO, retinal vein occlusion.

^†^ T-test.

^‡^Fisher’s exact test.

Table 3 shows the alterations of clinical data in patients with RVO by treatment. In total RVO, BCVA significantly improved from 0.40 at baseline to 0.22 at 1 month after anti-VEGF treatment (*P* < 0.001). Both CRT and CCT were significantly thinner at 1 month after treatment (*P* < 0.001 and *P* < 0.001, respectively). In branch RVO, BCVA also significantly improved from 0.35 at baseline to 0.15 at 1 month after treatment (*P* < 0.001). Both CRT and CCT were significantly thinner at 1 month after treatment (*P* < 0.001 and *P* = 0.003, respectively). In central RVO, BCVA significantly improved from 0.40 at baseline to 0.35 at 1 month after treatment (*P* = 0.004). CRT was significantly thinner at 1 month after treatment (*P* < 0.001), whereas CCT was not significant (*P* = 0.24).

**Table 3:** Alterations of clinical data in patients with RVO by treatment.

	Total RVO (n = 55)		*P* value	Branch RVO (n = 43)		*P* value	Central RVO (n = 12)		*P* value
	Baseline	1 month		Baseline	1 month		Baseline	1 month	
BCVA, median (IQR)	0.40 (0.15–0.52)	0.22 (0.05–0.40)	< 0.001^†^	0.35 (0.15–0.52)	0.15 (0.05–0.46)	< 0.001^†^	0.40 (0.28–0.57)	0.35 (0.21–0.40)	0.040^†^
CRT(μm), median (IQR)	477 (357–614)	224 (199–251)	< 0.001^†^	440 (343–586)	222 (196–244)	< 0.001^†^	574 (491–747)	239 (219–258)	<0.001^†^
CCT (μm), median (IQR)	238 (197–264)	215 (174–270)	0.001^†^	235 (180–259)	213 (160–267)	0.003^†^	257 (214–298)	242 (204–290)	0.24^†^

Abbreviations: IQR, interquartile range; BCVA, best-corrected visual acuity (LogMAR unit); CRT, central retinal thickness; CCT, central choroidal thickness; RVO, retinal vein occlusion.

^†^ Paired T-test.

**Fig 2:**
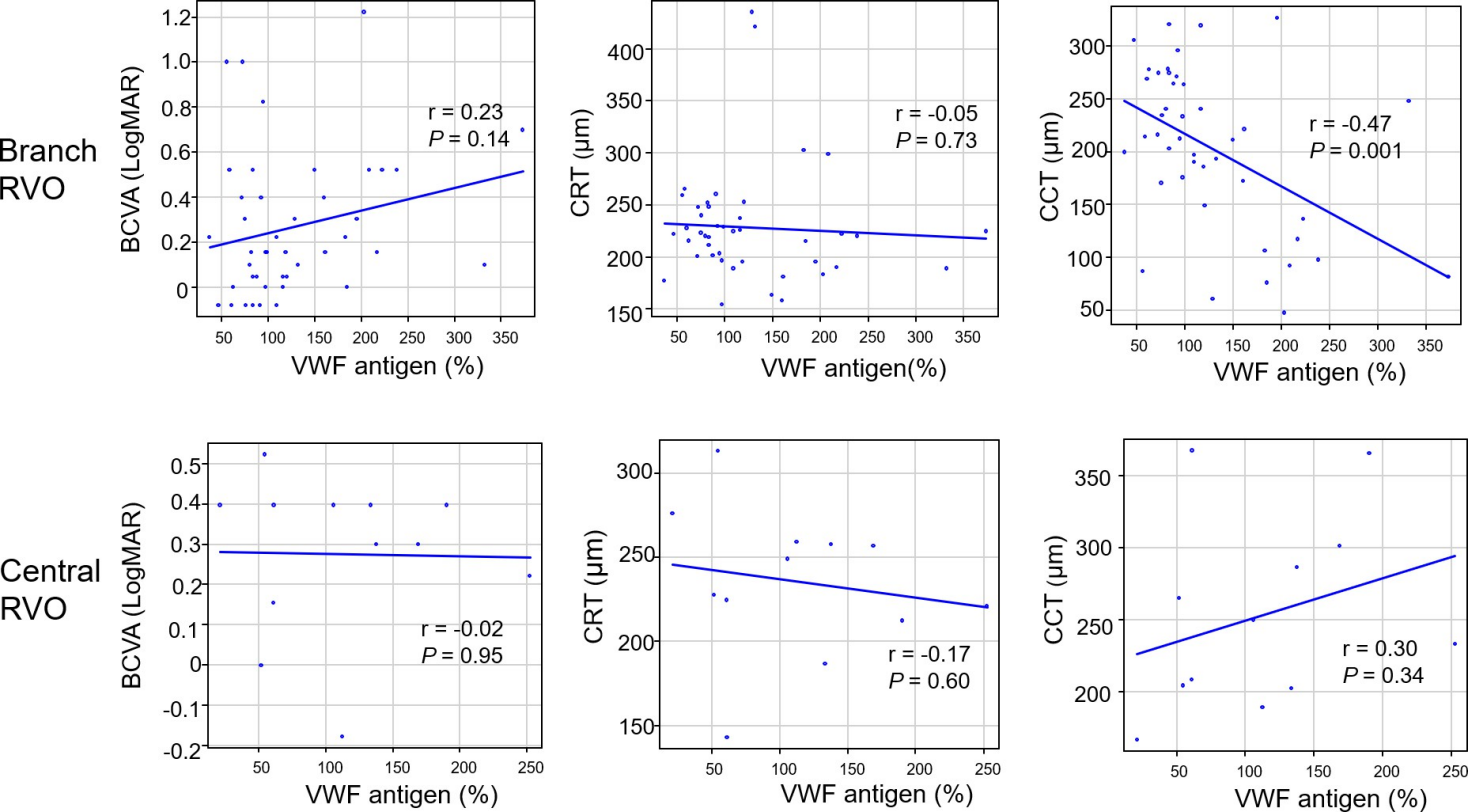
Correlations between VWF antigen and parameters (BCVA, CRT, and CCT) at 1 month after anti-VEGF treatment. In each graph, the horizontal axis presents the VWF antigen (%). BCVA, best-corrected visual acuity (LogMAR unit); CRT, central retinal thickness; CCT, central choroidal thickness; RVO, retinal vein occlusion.

Fig 2 shows the correlation between VWF antigen and each parameter at 1 month after anti-VEGF treatment. In branch RVO, VWF antigen was negatively correlated with CCT (r = −0.47, *P* = 0.001). In central RVO, no correlations were found in any of the categories.

**Fig 3:**
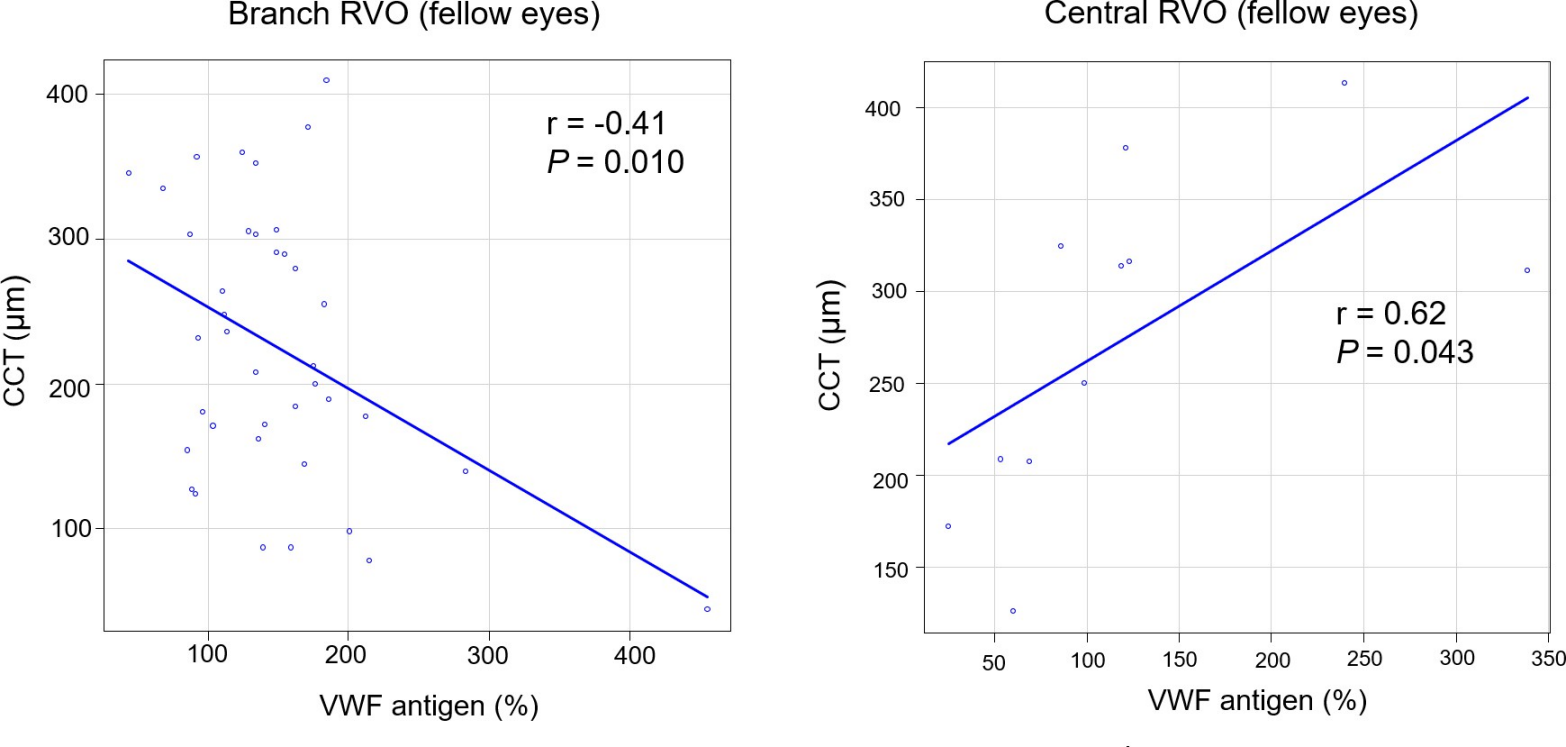
The correlation between VWF antigen and the CCT of fellow eyes at baseline. CCT, central choroidal thickness; RVO, retinal vein occlusion.

Fig 3 shows the correlation between VWF antigen and the CCT of fellow eyes at baseline. Five eyes were excluded owing to previous ocular disease. VWF antigen was negatively correlated with CCT (r = −0.41, *P* = 0.010) in branch RVO (n = 39) but positively correlated with CCT (r = 0.62, *P* = 0.043) in central RVO (n = 11).

**Fig 4:**
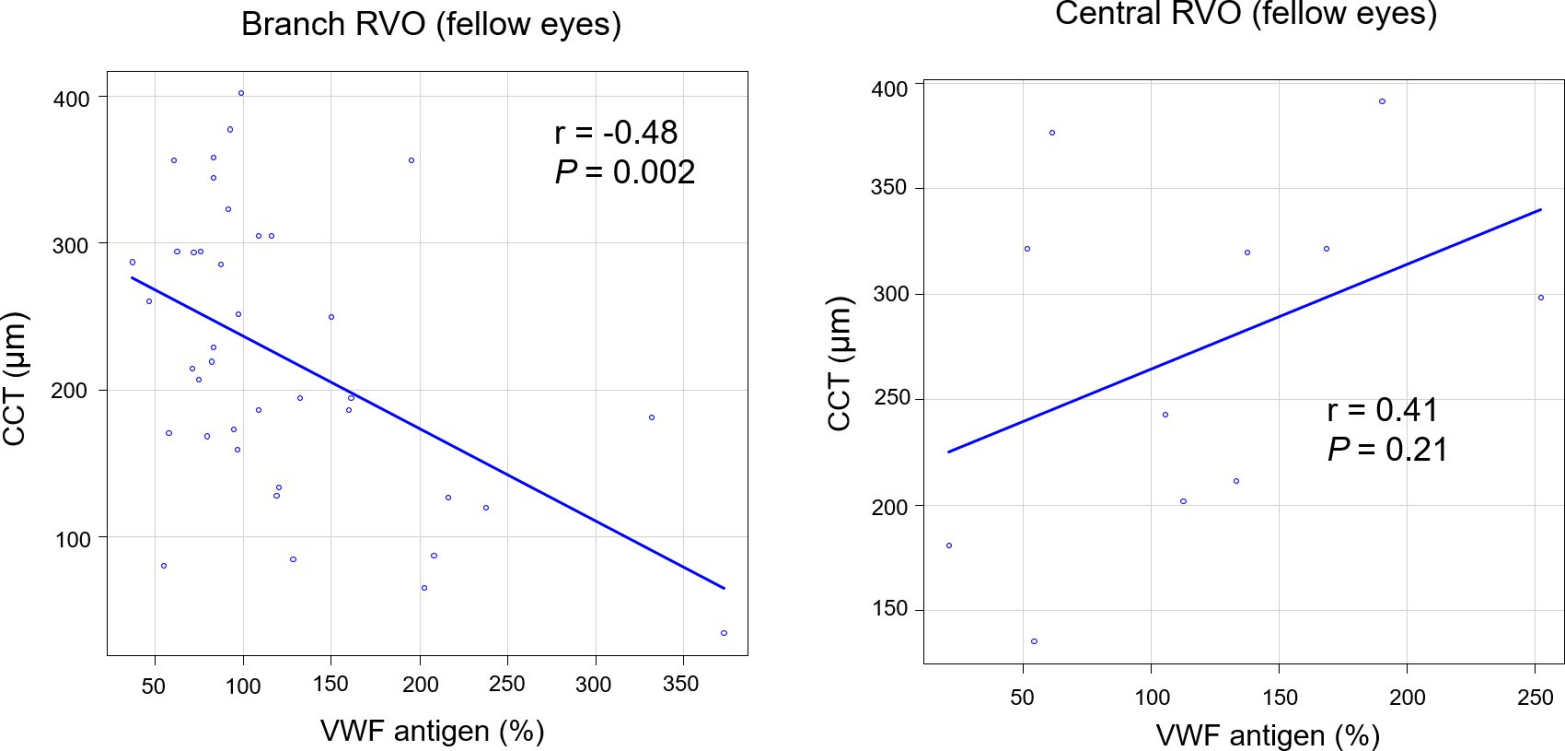
The correlation between VWF antigen and the CCT of fellow eyes at 1 month after anti-VEGF treatment. CCT, central choroidal thickness; RVO, retinal vein occlusion.

Fig 4 shows the correlation between VWF antigen and the CCT of fellow eyes at 1 month after anti-VEGF treatment. In branch RVO, VWF antigen was negatively correlated with CCT (r = −0.48, *P* = 0.002). In central RVO, there was no significant correlation.

**Fig 5:**
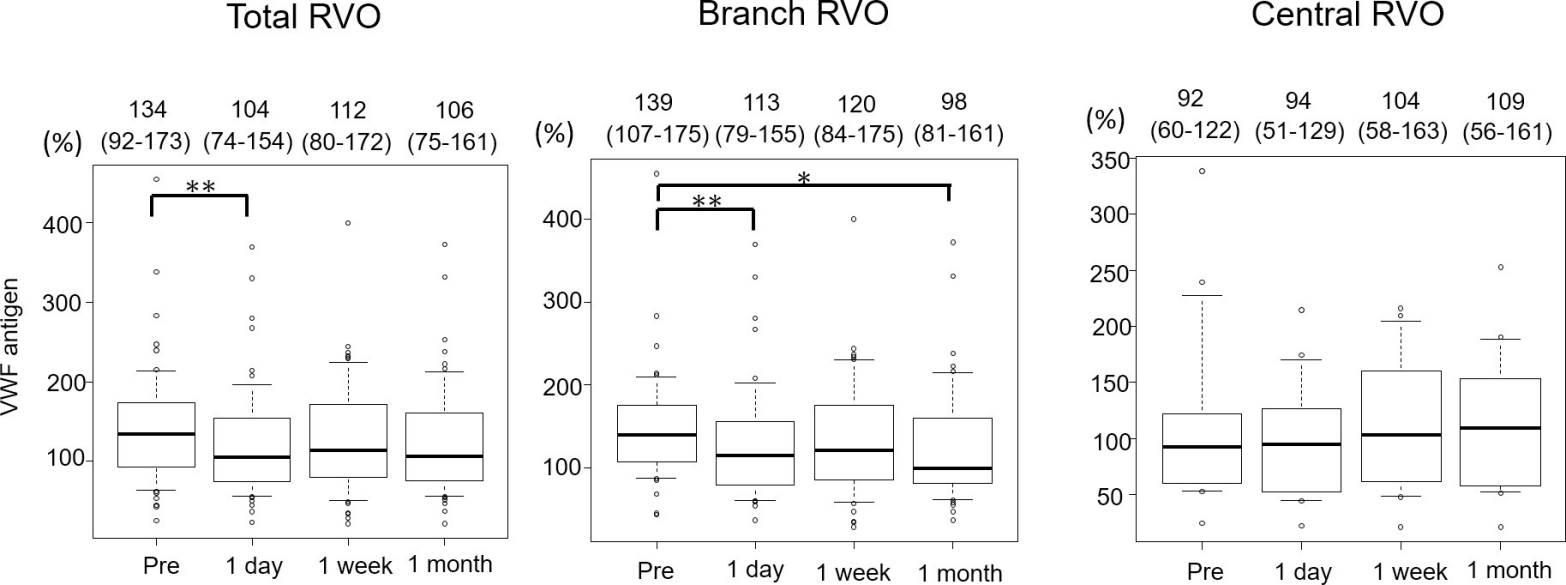
Alterations of VWF antigen after anti-VEGF treatment. Each of the values above the graph presents the median with the interquartile range. *< 0.05, **< 0.01. RVO, retinal vein occlusion.

Fig 5 shows the time course of VWF antigen in patients with total RVO, branch RVO, and central RVO. In total RVO, VWF antigen decreased significantly from 134% at baseline to 104% at 1 day after treatment (*P* = 0.002). In branch RVO, VWF antigen also showed a significant reduction from 139% at baseline to 113% at 1 day (*P* = 0.002) and 98% at 1 month (*P* = 0.030) after treatment. However, in central RVO, VWF antigen was not significant during this period. ADAMTS13 activity was not significant during the study period in all categories.

**Fig 6:**
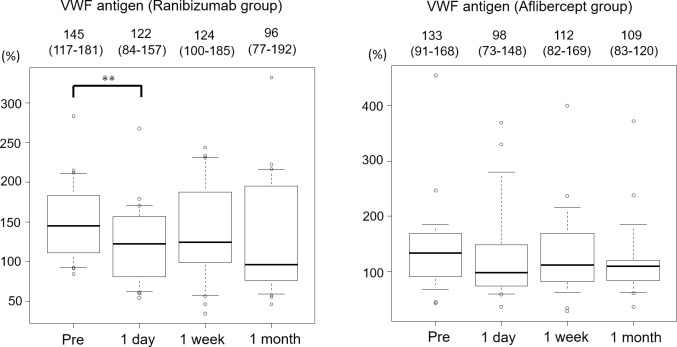
Alterations of VWF antigen in patients with branch RVO according to the administered drug. Each of the values above the graph presents the median with the interquartile range. *< 0.05, **< 0.01. RVO, retinal vein occlusion.

Fig 6 shows the time course of VWF antigen in patients with branch RVO according to the administered drug (ranibizumab or aflibercept). In the ranibizumab group (n = 22), VWF antigen decreased significantly from 145% at baseline to 122% at 1 day after treatment (P < 0.001). However, VWF antigen was not different in the aflibercept group (n = 21).

## Discussion

This study investigated the coagulation-related factors in patients with RVO at baseline and their alterations during treatment. According to our results, plasma VWF was not significant between the RVO group and age-matched controls at baseline. Several researchers have reported that plasma VWF increases not only in patients with hypertension and arteriosclerosis but also in healthy individuals with aging [28,29].

ADAMTS13 activity was significantly lower in central RVO than in branch RVO at baseline, but the number of dyslipidemia cases was significantly higher in central RVO. ADAMTS13 is mainly synthesized in hepatic stellate cells [2]. Thus, the liver cells in patients with dyslipidemia could be damaged by fat deposition, and production of ADAMTS13 could be decreased in patients with central RVO.

Although it was not significant, the VWF levels in branch RVO were higher than those in central RVO. Branch RVO is caused by a thrombus at the arteriovenous crossing point, where the retinal artery and vein share an outer sheath [30,31]; therefore, the arterial pressure is a direct cause of thrombosis. However, central RVO is caused by a thrombus in the central retinal vein, where it passes through the lamina cribrosa within the optic nerve. A recent study focusing on branch and central RVO has shown that atherosclerosis is more likely to lead to the development of branch RVO than central RVO [32]. The high levels of plasma VWF in our study suggest atherosclerosis in branch RVO.

We also compared VWF levels with each of the ophthalmologic parameters of RVO and found that VWF antigen was negatively correlated with CCT in branch RVO at baseline. This association between VWF antigen and CCT was also observed in fellow eyes, suggesting systemic VWF levels may affect choroidal circulation in patients with branch RVO. In contrast, VWF antigen was not significantly correlated with CCT in the affected eyes of patients with central RVO before or after treatment. These differences in results may suggest pathological differences between central and branch RVO. Moreover, central RVO has been reported to be accompanied by decreased ocular circulation and intense congestion [17], which may have influenced the association between VWF and choroidal thickness. The choroid, consisting of microcapillaries, and its structure can be observed noninvasively using optical coherence tomography. Several researchers have recently focused on the choroid as an indicator of systemic microcirculation [33,34], but the biochemical factors affecting the choroidal structure have not been well investigated. The studies examining the association between VWF and choroidal thickness are few; only Gifford et al. reported an inverse association between choroidal thickness and VWF in patients with liver cirrhosis [34].

Many previous reports demonstrated that choroidal thickness in patients with RVO decreases after intravitreal injection of anti-VEGF drugs [35]. Sakanishi et al. reported that the recurrence of cystoid macular edema was significantly low in cases with choroidal thinning after treatment [36]. Thus, CCT may reflect the treatment effect and prognosis in RVO. Moreover, VWF antigen was negatively correlated with CCT even at 1 month after anti-VEGF injection in branch RVO. The measurement of VWF antigen during treatment may be useful in evaluating the disease activity of branch RVO. VWF antigen also showed significant alterations under anti-VEGF treatment, especially in the ranibizumab group in branch RVO. Several researchers have previously proposed an association between VWF and VEGF [37,38].

VWF is secreted from endothelial-specific organelles called Weibel–Palade bodies that widely distribute it in systemic vessels [2]. VEGF activates exocytosis of Weibel–Palade bodies, resulting in the secretion of VWF, which activates VEGF-R2 signaling and regulates angiogenesis [39].

Pace et al. have recently reported decreased VWF plasma levels after anti-VEGF treatment (bevacizumab) in recurrent glioma [40]. This result was obtained with a relatively high dose (10 mg/kg) of the anti-VEGF drug administered intravenously. In the present study, we showed that plasma VWF concentrations can be altered even with small intravitreal doses (0.05 mL). The VWF levels in patients with branch RVO was significantly decreased in the ranibizumab group but not in the aflibercept group. Aflibercept is a recombinant protein composed of VEGF-R1 and 2 bound to the IgG-1 Fc portion. In contrast, ranibizumab is a monoclonal antibody fragment that inhibits the active isoform of VEGF-A [41]. The structural variations between aflibercept and ranibizumab may have resulted in the differences in release of VWF into the plasma via VEGF-R2.

This study has some limitations. First, this was a cross-sectional study with a relatively small sample size. A larger number of patients will be required for more reliable analyses. Second, we only assessed patients who received anti-VEGF drugs. It might be necessary to compare VWF levels in patients with and without treatment during the same time course. However, it is difficult to analyze untreated patients through frequent medical visits. Finally, we did not evaluate the long-term effects of anti-VEGF drugs for more than 1 month after intravitreal injection. A longer follow-up period is required to confirm these results.

## Conclusion

We performed prospective analyses of VWF in patients with RVO during treatment and found unique characteristics. Our findings suggest an association between VWF and CCT in branch RVO; thus, the measurement of VWF may be useful in evaluating disease activity and prognosis.
